# Hope and Distress Are Not Associated With the Brain Tumor Stage

**DOI:** 10.3389/fpsyg.2021.642345

**Published:** 2021-05-28

**Authors:** Simone Mayer, Stefanie Fuchs, Madeleine Fink, Norbert Schäffeler, Stephan Zipfel, Franziska Geiser, Heinz Reichmann, Björn Falkenburger, Marco Skardelly, Martin Teufel

**Affiliations:** ^1^Department of Neurology, University Hospital Carl Gustav Carus Dresden, Dresden, Germany; ^2^Department of Psychosomatic Medicine and Psychotherapy, University Hospital Tübingen, Tübingen, Germany; ^3^Department of Psychosomatic Medicine and Psychotherapy, LVR University Hospital Essen, Essen, Germany; ^4^Department of Psychosomatic Medicine and Psychotherapy, University Hospital Bonn, Bonn, Germany; ^5^Center for Neuro-Oncology, Comprehensive Cancer Center Tübingen-Stuttgart, University Hospital Tübingen, Tübingen, Germany; ^6^Department of Neurosurgery, University Hospital Tübingen, Tübingen, Germany; ^7^Department of Neurosurgery, District Hospital Reutlingen, Reutlingen, Germany; ^8^Section of Psycho-Oncology, West German Cancer Center (WTZ), University Hospital Essen, Essen, Germany

**Keywords:** depression, glioblastoma, brain tumor, meningioma, cancer, hope, distress, oncology

## Abstract

**Objective:**

Hopelessness and depression are strongly associated with suicidality. Given that physical and psychological outcomes can be altered with hope, hope is a therapeutic goal of increasing importance in the treatment of brain tumor patients. Moreover, it is not yet understood which factors affect the perception of hope in brain tumor patients. In addition, it remains uncertain whether lower-grade brain tumor patients suffer less from psycho-oncological distress than higher-grade brain tumor patients.

**Methods:**

Neuro-oncological patients were examined perioperatively with the Distress Thermometer (DT) and the Herth Hope Index (HHI). In addition, psychological comorbidities (anxiety GAD-2, depression PHQ-2) and an assessment of general psycho-oncological distress were recorded.

**Results:**

Sixty-six brain tumor patients were included (median age 53 years, 35% higher-grade brain tumors, i.e., WHO grade III/IV). No differences between higher- and lower-grade brain tumor patients were observed for general psycho-oncological distress and hope. However, higher-grade brain tumor patients showed a significantly higher level of depression (*p* ≤ 0.001) and more negative expectations regarding therapeutic success (*H* = 4.873, *p* ≤ 0.050). The extent of depression correlated negatively with hope.

**Conclusion:**

Unexpectedly, higher-grade brain tumor patients remained as hopeful as lower-grade brain tumor patients despite the devastating diagnosis, higher levels of depression, and a worse expectation of therapeutic success. Conversely, lower-grade brain tumor patients experience as much psycho-oncological distress as patients with a higher-grade brain tumor, underpinning the imperative need for comprehensive psycho-oncological screening. For all brain tumor patients, considering hope is important to avoid suicides resulting from hopelessness and depression.

## Introduction

Hope is inherent in human nature, i.e., it is mainly determined by innate personality traits; but the disease process of cancer can have an impact on an individual’s level of hope (Corn et al., 2020). Hope can be fostered by psychotherapeutic treatment (Rustøen et al., 2011) and should therefore be considered as a beneficial therapeutic target to improve quality of life in cancer patients (Corn et al., 2020).

Quality of life, self-esteem, coping, adjustment to an illness, well-being, satisfaction with information received, and relationships are important factors for the feeling of hope (Vellone et al., 2006). In consequence, aspects of hope, such as positive expectations and optimism, can influence psychological and physical outcomes (Leedham et al., 1995; Duggal et al., 2016).

It is expected that hope depends on the tumor stage since patients with an early stage diagnosed tumor feel more hope than patients with an advanced stage or recurring tumor (Acquaye et al., 2016). There is a tendency toward increased psychological distress in more aggressive tumors with worse prognosis (Trad et al., 2015). But even patients with a lower-grade brain tumor have a high psychological burden. More than 10 years after surgery they still experience an impairment of their quality of life (Nassiri et al., 2019). Conversely, patients receiving palliative treatment do not differ significantly in their experience of hope from patients receiving curative therapy (Sanatani et al., 2008). Therefore, it cannot be conclusively determined whether patients with a higher-grade brain tumor experience more hope than patients with a lower-grade brain tumor. However, there is no doubt that hope is clinically relevant because it may help patients to improve their health behavior, accept help from caregivers, and stay in treatment, which contributes to longer survival (Aspinwall and Tedeschi, 2010; Mahendran et al., 2016). In some studies, a direct effect of hope on survival has also been investigated, but this remains controversial (Nagano et al., 2006; Nakaya et al., 2008).

Depression, anxiety, and general psycho-oncological distress have a negative impact on quality of life in cancer patients (Mehnert et al., 2010, 2018). These factors are assumed to correlate with feelings of hope in brain tumor patients. The recognition of depression and mood disorders, which are frequent in brain tumor patients (Bunevicius et al., 2013b), is immensely important in long-term cancer care (Mitchell et al., 2011). The distress burden of depression remains high during the course of the brain tumor disease (Renovanz et al., 2019), which may lead to a poorer quality of life associated with increased mortality (Gathinji et al., 2009). Depression and hopelessness are associated with the construct of demoralization (Ignatius and De La Garza, 2019). Demoralization, in turn, evokes a desire for hastened death or suicide (Rzeszut and Assael, 2021). Because suicide rates are twice as high in cancer patients as in the general population, it is important to recognize the risk factors depression and demoralization associated with hopelessness (McFarland et al., 2019).

As of today, despite quite a few studies, findings on the experience of hope at different stages of cancer are inconsistent. Given the potential of hope to alter oncologic outcomes in cancer patients and the opportunity to improve quality of life, there is a growing demand for more research in this area (Corn et al., 2020).

The aim of this study was to investigate if patients with a higher-grade brain tumor have less hope and more general psycho-oncological distress than patients with a lower-grade brain tumor. In addition, we compared patients with a lower- vs. higher-grade brain tumor with respect to psycho-oncological issues and determined the factors that are associated with the experience of hope.

## Methods

A cross-sectional, single-center study was conducted between September 2015 and November 2016 with brain tumor patients of the Neurosurgery Department of Tübingen University Hospital. Inclusion criteria were patients who understood the local language and presented with any kind of brain tumor that requires a biopsy or craniotomy. Exclusion criteria were severe problems with the German language, severe physical problems (e.g., sedation, concentration deficits), illiteracy, or severe difficulties in usage of a tablet PC. Seventy-one patients were consecutively screened, with five patients excluded (one brain metastasis in renal cell carcinoma, two Adenoma of the adenohypophysis, one unclear mass without biopsy, one schizophrenia). Sixty-six patients were assessed and included in the analysis. The patients gave electronic informed consent and predominantly completed the questionnaire on a tablet PC in the hospital. The patients were accustomed to using a tablet PC (Schäffeler et al., 2013). The assessment took place perioperatively (61 out of 66 patients were interviewed the day before surgery, 5 out of 66 patients were interviewed 4–6 days after surgery).

Ethics approval was obtained beforehand from the local ethics committee of the Medical Faculty at the University of Tübingen (reference number 602/2014BO2).

The assessment for socio-demographic data included marital status, current living situation, highest level of education, and working state. The assessment for mental history included questions about psychotropic medication and psychotherapeutic treatment at present or past. The patients were classified into lower-grade (WHO stage I/II) and higher-grade (WHO stage III/IV) brain tumor patients (Table 1). For 56 out of 66 patients it was the first brain surgery while 10 out of 66 patients received a recurrent resection (Table 2).

**TABLE 1:** Classification of patients in the respective WHO stage group (*N* = 66).

**Tumor**	**Total number N (%)**		**Grouping**
Meningothelial meningioma^†^	16	(24.2%)	WHO stage I/II
Transitional meningioma^†^	6	(9.1%)	WHO stage I/II
Fibrous meningioma^†^	5	(7.6%)	WHO stage I/II
Atypical meningioma^†^	5	(7.6%)	WHO stage I/II
Oligodendroglioma^†^	4	(6.1%)	WHO stage I/II
Diffuse astrocytoma^†^	2	(3.0%)	WHO stage I/II
Choroid meningioma^†^	1	(1.5%)	WHO stage I/II
Pilocytic meningioma^†^	1	(1.5%)	WHO stage I/II
Oligoastrocytoma^†^	1	(1.5%)	WHO stage I/II
Gangliocytoma^†^	1	(1.5%)	WHO stage I/II
Ganglioglioma^†^	1	(1.5%)	WHO stage I/II
Glioblastoma	19	(28.8%)	WHO stage III/IV
Anaplastic Astrocytoma	3	(4.5%)	WHO stage III/IV
Other WHO stage III	1	(1.5%)	WHO stage III/IV

*^†^Meningiomas and WHO grade I/II tumors were grouped together because T-tests showed no significant differences in general psycho-oncological distress (depression p = 0.35, anxiety p = 0.73, distress p = 0.09, and hope p = 0.25), although they are treated differently and have a different prognosis.*

**TABLE 2:** Sample description.

**Feature**	**Lower-grade brain tumors (WHO Stage I/II) *N* = 43 (65.2%)**	**Higher-grade brain tumors (WHO stage III/IV) *N* = 23 (34.8%)**	**Total *N* = 66 (100%)**	***p*-value**
**Socio-demographic data**				
Sex, female	30 (69.8%)	8 (34.8%)	38 (57.6%)	**0.006 ^*C*^**
Age (years)	M 53.7 ± 11.1	M 51.5 ± 14.9	M 53.0 ± 12.5	0.088 ^*T*^
***Marital status***				
With partner	32 (74.4%)	19 (82.6%)	51 (77.3%)	
Single	3 (7.0%)	3 (13.0%)	6 (9.1%)	0.226 ^*C*^
Others	8 (18.6%)	1 (4.3%)	9 (13.6%)	
***Life and living situation***				
With family	33 (76.7%)	21 (91.3%)	54 (81.8%)	
Alone	8 (18.6%)	1 (4.3%)	9 (13.6%)	0.269 ^*C*^
Others	2 (2.7%)	1 (4.3%)	3 (4.5%)	
***Educational level***				
School (≥10 years)	5 (11.6%)	2 (8.7%)	7 (10.6%)	
School (<10 years)	25 (58.1%)	17 (73.9%)	42 (63.6%)	0.610 ^*C*^
University	11 (25.6%)	3 (13.0%)	14 (21.2%)	
Others	2 (4.7%)	1 (4.3%)	3 (4.5%)	
***Working state***				
Working	31 (72.1%)	16 (69.6%)	47 (71.2%)	
Not working	12 (27.9%)	7 (30.4%)	19 (28.8%)	0.826 ^*C*^
**Psycho-oncological data**				
PHQ-2	43 (100%)	23 (100%)	66 (100%)	0.12 ^*U*^
	M 1.47 ± 1.44	M 1.96 ± 1.43	M 1.64 ± 1.44	
	min 0; max 6	min 0; max 4	min 0; max 6	
	Mdn 1.0	Mdn 2	Mdn 1.5	
	IQR 0–2	IQR 1–3	IQR 0–2	
PHQ-2 ≥ 3	5 (11.6%)	9 (39.1%)	14 (21.2%)	**0.009 ^*C*^**
GAD-2	43 (100%)	23 (100%)	66 (100%)	
	M 1.74 ± 1.31	M 2.52 ± 2.09	M 2.02 ± 1.65	
	min 0; max 6	min 0; max 6	min 0; max 6	0.20 ^*U*^
	Mdn 1.0	Mdn 2	Mdn 2	
	IQR 1–2	IQR 1–4	IQR 1–3	
GAD-2 ≥ 3	9 (20.9%)	10 (43.5%)	19 (28.8%)	0.054 ^*C*^
HHI	42 (97.3%)	20 (87.0%)	62 (93.9%)	
	M 42.5 ± 4.75	M 42.2 ± 4.95	M 42.4 ± 4.78	
	min 24; max 48	min 28; max 48	min 24; max 48	0.922 ^*U*^
	Mdn 43.5	Mdn 44.5	Mdn 44.0	
	IQR 40–46	IQR 39–45	IQR 36–40	
HHI missing	1 (2.3%)	3 (13.0%)	4 (6.1%)	
DT	43 (100%)	23 (100%)	66 (100%)	
	M 4.93 ± 2.44	M 5.87 ± 2.36	M 5.26 ± 2.44	
	min 1; max 10	min 2; max 10	min 1; max 10	0.137 ^*T*^
	Mdn 5	Mdn 6	Mdn 5	
	IQR 3–7	IQR 4–8	IQR 3–7	
DT ≥ 5	25 (58.1%)	16 (69.6%)	41 (62.1%)	0.431 ^*F*^
Physical problems	35 (81.4%)	15 (65.2%)	50 (75.8%)	0.144 ^*C*^
Family problems	5 (11.6%)	2 (8.7%)	7 (10.6%)	0.534 ^*F*^
Emotional problems	34 (79.1%)	18 (78.3%)	52 (78.8%)	0.939 ^*C*^
Spiritual problems	1 (2.3%)	2 (8.7%)	3 (4.5%)	0.276 ^*F*^
Practical problems	5 (11.3%)	7 (30.4%)	12 (18.2%)	0.059 ^*C*^
**Clinical data**				
***Tumor localization***				
Right	21 (48.8%)	12 (52.2%)	33 (50%)	
Left	18 (41.9%)	9 (39.1%)	27 (40.9%)	0.967 ^*C*^
Not localizable	4 (9.3%)	2 (8.7%)	6 (9.1%)	
**Disease duration** (months)	M 33.2 ± 55.7	M 8.17 ± 16.6	M 24.5 ± 47.3	
	min. 0; max 246	min 0; max 74	min. 0; max. 246	**0.001 ^*U*^**
	Mdn 8.0	Mdn 1.0	Mdn 6.0	
	IQR 2–34	IQR 0–6	IQR 1–26.25	
***Surgical procedure***				
Only biopsy	2 (4.7%)	5 (21.7%)	7 (10.6%)	**0.045 ^*F*^**
Complete resection	36 (83.7%)	13 (56.5%)	49 (74.2%)	**0.021 ^*F*^**
Recurrent resection	10 (23.3%)	9 (39.1%)	19 (28.8%)	0.245^ F^
***Psychotherapeutic treatment***				
Never	32 (74.4%)	16 (69.9%)	48 (72.7%)	
Current	2 (4.7%)	2 (8.7%)	4 (6.1%)	0.795 ^*C*^
Former	9 (20.9%)	5 (21.7%)	14 (21.2%)	
***Psychotropic medication***				
Never	37 (86.0%)	18 (78.3%)	55 (83.3%)	
Every day	3 (7.0%)	5 (21.7%)	8 (12.1%)	0.110^ C^
Occasional	3 (7.0%)	0 (0%)	3 (4.5%)	

*PHQ-2, Patient Health Questionnaire two items; GAD-2, Generalized Anxiety Disorder two items; HHI, Herth-Hope Index; DT, Distress Thermometer; IQR, Interquartile Range; M, Mean; Mdn, Median; C, Chi-squared test; T, T-test; U, Mann-Whitney U test; F, Fisher’s exact test. The bold values represent a statistical significance ≤ 0.05*

### Assessment Instruments

#### Distress Thermometer (DT)

Distress Thermometer (DT) is a scale with values from 0 to 10. Zero means “no distress” and 10 means “extreme distress” (Roth et al., 1998; National Comprehensive Cancer Network, 1999, 2003). The patients indicate which value best describes their level of distress during the previous week. In addition, the DT comprises a problem list including 34 items on practical, emotional, family, spiritual, and physical problems (Mehnert et al., 2006; Rapp et al., 2018). A DT cut-off ≥ 5 is considered appropriate (Lambert et al., 2014). The DT is a valid and reliable screening instrument with 83% sensitivity and 68% specificity (Donovan et al., 2014).

#### Generalized Anxiety Disorder-2 (GAD-2)

Generalized Anxiety Disorder-2 (GAD-2) is a short form measurement with two questions for screening generalized anxiety disorder (Spitzer et al., 2006). From the summation of both questions, a scale value in the range of 0–6 points is calculated. Higher scores correspond to higher anxiety. A value ≥ 3 is regarded as a serious indicator of pathological anxiety (Spitzer et al., 2006; Kroenke et al., 2007). The GAD-2 performs well (area under the curve, 0.80–0.91) as a screening tool for anxiety disorders (Kroenke et al., 2007) and is well-suited for brain tumor patients, as demonstrated for neurological patients with acceptable internal consistency (Cronbach’s α = 0.77) (Hughes et al., 2018) and head and neck cancer patients with good internal consistency (Cronbach’s α = 0.87) (Hammermüller et al., 2021).

#### Patient Health Questionnaire-2 (PHQ-2)

Patient Health Questionnaire-2 (PHQ-2) is a short measurement consisting of two questions to detect depression (Löwe et al., 2005). The combined value of both questions (generated by addition) ranges from 0 to 6 with higher values indicating stronger depressive symptoms. A cut-off value of ≥ 3 is the optimum for screening a depression. The PHQ-2 shows a sensitivity of 87% and a specificity of 78% for major depressive disorder (Löwe et al., 2005). Preliminary results show that depression can be successfully identified in brain tumor patients by using the PHQ-2, which has moderate internal consistency (Cronbach’s α = 0.68) (Bunevicius et al., 2013a).

#### Herth-Hope-Index (HHI)

Herth-Hope-Index (HHI) is a proven and practical instrument in research and clinical setting for the assessment of hope and shows a good reliability and validity with12 items on a 4-point Likert scale ranging from 12 to 48 (higher sores indicate more hope) (Herth, 1992). The German version of the HHI used in this study shows high practicability, good internal consistency (Cronbach’s α = 0.82), and solid concurrent validity, especially for cancer patients (Geiser et al., 2015).

#### Questionnaire for Coping Parameters

Questionnaire for coping parameters was designed for this study. The questionnaire consists of the following six 10-point Likert-scale aspects: (*i*) expectations of therapeutic success, (*ii*) expectations of side effects, (*iii*) former resilience rating, (*iv*) current state of mental health, (*v*) ability to handle the disease, and (*vi*) social environment support.

### Statistical Analyses

**FIGURE 1:**
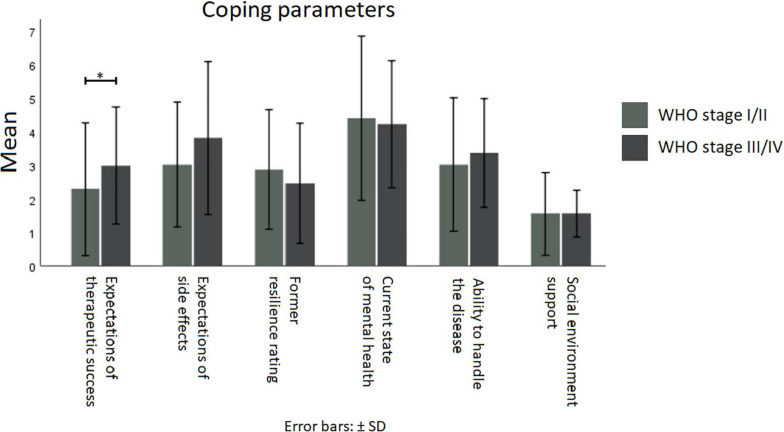
Means of coping parameters for WHO stages I/II (light gray) vs. stages III/IV (dark gray). Kruskal-Wallis test showed no differences between lower and higher WHO stages, except for the expectations of therapeutic success (*H* = 4.873, *p* ≤ 0.050, *N* = 66). *p ≤ 0.05.

Statistical analysis was performed using statistical program for social sciences (SPSS) version 25 (IBM New York). Differences between the two dichotomous WHO stage groups (WHO stage I/II vs. WHO stage III/VI), were analyzed by means of Chi-square test for categorical variables, Mann-Whitney *U* test for metric scaled non-normally distributed variables, *T*-test for metric-scaled normally distributed variables, and Fisher’s exact test for small sample sizes (Table 2). In addition, the coping parameter differences between the two dichotomous WHO stage groups were investigated by means of the Kruskal-Wallis test (Figure 1). To identify parameters that are associated with hope in patients with a brain tumor, linear logistic regression was used. Aligned with the study sample’s *N* = 66 patients, the three variables depression, anxiety, and general psycho-oncological distress which had a statistical influence on univariate analysis were considered (Table 3). A *p*-value less than 0.05 was regarded as statistically significant.

**TABLE 3:** Multivariate regression analysis (inclusion method) for determining factors associated with the perception of hope (*N* = 61).

	**HHI**			
**Variables**	**B**	**SE**	**β**	***p*-value**
PHQ-2	–1.44	0.48	–0.43	<0.01
GAD-2	–0.49	0.42	–0.17	0.25
DT	–0.11	0.25	–0.06	0.66
R^2^	0.113			

*HHI, Herth-Hope Index; PHQ-2, Patient Health Questionnaire two items; GAD-2, Generalized Anxiety Disorder two items; DT, Distress Thermometer.*

## Results

This study includes the complete data sets from 66 patients except the HHI which was completed by 62 patients. Apart from sex, socio-demographic data between the WHO stage groups were comparable. Patients with a lower-grade brain tumor were more frequently female (*p* = 0.006) compared with higher-grade brain tumor patients (Table 2).

### Tumor-Specific Data

Compared with higher-grade brain tumor patients, patients with a lower-grade brain tumor suffered from a longer course of disease (*p* = 0.001) and more often received a complete resection (*p* = 0.021) rather than a biopsy (*p* = 0.045). Regarding the lateral location of the tumors, no notable difference between the groups was detected (Table 2).

### Psycho-Oncological Data

The lion’s share of patients never received psychotherapeutic treatment (72.7%) or psychotropic medication (83.3%). Apart from depression, there were no differences in other psycho-oncological data, especially not in hope (*p* = 0.922), general psycho-oncological distress (*p* = 0.137), and anxiety (*p* = 0.054). Noteworthy, patients with a higher-grade brain tumor were more often depressed (*p* = 0.009). The observed overall prevalence of depression (21.2%) and anxiety (28.8%) are in line with previous studies (Arnold et al., 2008; Huang et al., 2017). Due to the DT cut-off ≥ 5, the patient’s distress levels in this study are higher compared with previously studies published which use ≥ 6 as DT cut-off (Liu et al., 2018).

According to the DT problem list, about three out of four patients reported emotional (78.8%) and physical problems (75.8%). The most represented within emotional problems were sadness (50%), fear (40.9%), and loss of interest (39.4%). Getting around (36.4%), nausea (31.8%), sleep (33.3%), and sexual problems (25.8%) were the most frequently reported physical problems. Of a lesser mention were practical (18.2%), family (10.6%), and spiritual problems (4.5%).

For disease duration, no differences in over threshold psycho-oncological data (anxiety *p* = 0.18 ^*C*^, depression *p* = 0.55 ^*C*^, general psycho-oncological distress *p* = 0.32 ^*C*^, and hope 0.81 ^*C*^) were observed using the median split of 6 months.

There are also no differences in gender in over threshold psycho-oncological data (anxiety *p* = 1.00 ^*F*^, depression *p* = 0.56 ^*F*^, general psycho-oncological distress *p* = 0.61 ^*F*^, and hope 1.00 ^*F*^).

### Coping Factors and Hope

The HHI of this study showed an average of 42.4 out of 48 points (Table 2). Higher- and lower-grade brain tumor patients showed similar hope levels. This demonstrates that brain tumor patients’ hope levels are not significantly different compared with tumor patients in general (Geiser et al., 2015). Regarding the expectation of therapeutic success, a statistically significant difference between higher (*n* = 23) and lower WHO tumor stages (*n* = 43) was observed. Patients with a higher tumor stage showed lower expectations of therapeutic success (Kruskal-Wallis test, *H* = 4.873, *p* ≤ 0.050). Apart from that, no further differences in coping factors were identified (Figure 1).

### Parameters Associated With Hope

Depression, without doubt, was identified as a negative factor influencing hope. The PHQ-2 score shows a unique statistically significant contribution (*p* < 0.01) in the multivariate linear regression analysis (R^2^ is 0.340 and the entire model is statistically significant with *p* < 0.001). Cases with missing data in the HHI were excluded from the model. The dependent variable was the total value in the HHI. In contrast, general psycho-oncological distress and anxiety has no statistically significant contribution.

## Discussion

The data obtained in this study revealed that patients with a higher-grade brain tumor have just as much hope as lower-grade brain tumor patients despite a higher level of depression and a worse expectation of therapeutic success. This is all the more surprising because the evaluation also revealed that depression is negatively correlated with the feeling of hope. Contrary to the prevailing assumption, no differences in general psycho-oncological distress between higher- and lower grade brain tumor patients was observed. Despite over threshold values in the general psycho-oncological distress assessment, perioperatively, both groups experienced similar high levels of hope compared with the general population (Wahl et al., 2004).

In the group with the lower-grade brain tumors the female sex was more frequently represented. This finding is consistent with the literature as the increased occurrence of meningiomas has been hypothesized by female hormones (Wiemels et al., 2010). In general public, the distribution of the tumor entities of glioblastomas and meningiomas is similar (Wiemels et al., 2010; Crocetti et al., 2012). The other statistical differences in treatment and clinical characteristics, namely more complete resections and longer treatment durations of lower-grade brain tumor patients as well as more biopsies in patients with higher-grade brain tumors, result from the respective treatment guidelines and the pathophysiological tumor characteristics (Marosi et al., 2008).

### Strengthening Hope—Reducing Depression

Hope is an important factor in the treatment of cancer patients (Ho et al., 2010). The feeling of hope is essential for a viable doctor-patient relationship and necessary in the mental processing of a life-threatening disease (Clayton et al., 2008; Werner and Steihaug, 2017).

We have demonstrated that in the present sample of patients with brain tumors, depression is negatively correlated with the feeling of hope, which is consistent with previous results (Thimm et al., 2013).

It is to be strongly expected that this finding will have a negative impact on the quality of life (Szramka-Pawlak et al., 2014). Hopelessness has been discussed as a factor for suicidal ideation (Liu et al., 2015; Robinson et al., 2015; Ribeiro et al., 2018) and traumatic brain injuries are just an additional risk factor (Fazel et al., 2014; Fralick et al., 2019). Hypothetically, also neurosurgical interventions should be added to the list. In particular, the occurrence of cognitive inhibition deficits as a consequence of altered brain activation, may facilitate suicidal acts (Richard-Devantoy et al., 2016). Suicidal ideations reported from brain tumor patients are not more frequent compared to the general population (Forkmann et al., 2012; Pranckeviciene et al., 2016). However, to this day, the consequences of hopelessness on this matter are not explicitly investigated. The effect of hope on suicidal ideation and its interaction with depression should be further investigated in particular in brain tumor patients. Fostering quality of life by supporting a sense of hope in all tumor stages and grades remains a central task for both, oncology and psycho-oncology.

It is expected that higher-grade brain tumors patients are more depressed and anxious than those with a lower-grade brain tumor. Much to our astonishment, no significant difference in distress levels between the groups was observed. The direct association of brain lesions with depression (Richard-Devantoy et al., 2016) is one possible explanation in particular because wide spreading of glioblastomas tumor cells is a typical characteristic of the course of the disease (Vollmann-Zwerenz et al., 2020).

In this study, both groups do not significantly differ in both, hope and distress. For the only minor differences in hope there are three likely causes, (*i*) higher-grade brain tumor patients deny their devastating prognosis (Williams et al., 2015), (ii) the measurement is not specific enough to detect even small differences in hope levels between higher- and lower-grade brain tumor patients (Redlich-Amirav et al., 2018; Rustøen et al., 2018), and (*iii*) hope with its dispositional aspects (Demirli et al., 2015) is a coping mechanism (Clayton et al., 2005). Hope as a coping mechanism may even increase when the prognosis is poor (Palacio and Arias, 2018). For the insignificant differences in distress, two causes seem likely, (*i*) the diagnosis “brain tumor” overshadows the patients’ assessments of the distress thermometer, and (*ii*) the denial and hope for cure protects against devastating distress levels even when the prognosis is disastrous. Consequently, in future psycho-oncological screenings, we urge to also pay the needed attention to patients with lower-grade brain tumors.

### Hope Springs Eternal in Patients With a Brain Tumor

The patient group with a higher-grade brain tumor showed a more negative expectation regarding the therapeutic success, probably due to a comprehensive information about the worse course of the disease. Expectedly, high HHI values are found when the disease severity is low (e.g., no metastases, curative therapy) (Wakiuchi et al., 2015; Acquaye et al., 2016; Mahendran et al., 2016). Contrary to the expectation, however, also cancer patients with an unfavorable prognosis showed high HHI values which indicates that hope is not necessarily related to tumor entities or recurrence (Ballard et al., 1997; Felder, 2004). Also, in this instance, it seems plausible that hope grows also when the news is bad, provided it is sustained by trust in the treating team. For both, hope and quality of life, the paradox presumably applies that high burden can even increase hope or quality of life, because there is a shift in the disease appraisal (Albrecht and Devlieger, 1999).

The study is limited by the fact that the patient assessment was carried out perioperatively for logistical reasons. It goes without saying that it is preferable to survey patients at the time of diagnosis. Also, there is an imbalance between male (30.2%) and female (69.8%) patients in our cohort, caused by the more frequent occurrence of meningiomas in women. However, the imbalance is put into perspective in a subgroup analysis, which shows no gender dependent effect in the psycho-oncology data and the extent of hope. Because of the cross-sectional study design, no predictive statements can be made. The relatively small sample size could affect the validity and generalizability of the study. Longitudinal studies with a larger sample size allow predictive and valid statements.

## Conclusion

As expected, also in our study, depression is substantially pronounced in higher-grade brain tumor patients. However, when it comes to hope, anxiety, and distress, no significant differences between higher- and lower-grade brain tumor patients were observed. Both, higher- and lower-grade brain tumor patients feel much hope, but at the same time also much anxiety and distress. The current investigation contributes with new findings that patients with lower-grade brain tumors also suffer from pathological anxiety and general psycho-oncological distress. There is a correlation between lower hope levels and depression. Therefore, beyond routine psycho-oncological screening, hope should be considered as an important factor in the treatment of brain tumor patients. We emphasize that psycho-oncological screenings, as they are performed in particular in the higher-grade brain tumor patient group, also should be implemented supposedly benign diseases like a meningioma.

## Data Availability Statement

Data are available upon request. Requests should be directed to the corresponding author.

## Ethics Statement

The studies involving human participants were reviewed and approved by the Ethics Committee of the Medical Faculty at the University of Tübingen. The patients/participants provided their written informed consent to participate in this study.

## Author Contributions

SM: conception, data acquisition, data analysis, interpretation of the results, and writing of the manuscript. MS and SF: assistance in data acquisition, conception, and contributions to the writing of the manuscript. MF: assistance in data analysis and interpretation of the results. BF, NS, SZ, FG, and HR: contributions to the writing of the manuscript and interpretation of the results. MT: conception, data analysis, interpretation of the results, and contributions to the writing of the manuscript. All authors contributed to the article and approved the submitted version.

## Conflict of Interest

The authors declare that the research was conducted in the absence of any commercial or financial relationships that could be construed as a potential conflict of interest.
